# Phenotypic and genomic comparison of Mycobacterium aurum and surrogate model species to Mycobacterium tuberculosis: implications for drug discovery

**DOI:** 10.1186/s12864-017-3924-y

**Published:** 2017-07-13

**Authors:** Amine Namouchi, Mena Cimino, Sandrine Favre-Rochex, Patricia Charles, Brigitte Gicquel

**Affiliations:** 1 Centre for Ecological and Evolutionary Synthesis, Department of Biosciences, University of Oslo, P.O. Box 1066, Blindern NO-0316, Oslo, Norway; 2 Unit of Mycobacterial genetics, Institut Pasteur, 25-28, rue du Docteur Roux, 75724 Paris, Cedex 15 France

**Keywords:** Drug discovery, Tuberculosis, Mycobacterium Aurum, Comparative genomics, Drug resistance, Whole genome sequencing

## Abstract

**Background:**

Tuberculosis (TB) is caused by *Mycobacterium tuberculosis* and represents one of the major challenges facing drug discovery initiatives worldwide. The considerable rise in bacterial drug resistance in recent years has led to the need of new drugs and drug regimens. Model systems are regularly used to speed-up the drug discovery process and circumvent biosafety issues associated with manipulating *M. tuberculosis*. These include the use of strains such as *Mycobacterium smegmatis* and *Mycobacterium marinum* that can be handled in biosafety level 2 facilities, making high-throughput screening feasible. However, each of these model species have their own limitations.

**Results:**

We report and describe the first complete genome sequence of *Mycobacterium aurum* ATCC23366, an environmental mycobacterium that can also grow in the gut of humans and animals as part of the microbiota. This species shows a comparable resistance profile to that of *M. tuberculosis* for several anti-TB drugs. The aims of this study were to (i) determine the drug resistance profile of a recently proposed model species, *Mycobacterium aurum,* strain ATCC23366, for anti-TB drug discovery as well as *Mycobacterium smegmatis* and *Mycobacterium marinum* (ii) sequence and annotate the complete genome sequence of this species obtained using Pacific Bioscience technology (iii) perform comparative genomics analyses of the various surrogate strains with *M. tuberculosis* (iv) discuss how the choice of the surrogate model used for drug screening can affect the drug discovery process.

**Conclusions:**

We describe the complete genome sequence of *M. aurum*, a surrogate model for anti-tuberculosis drug discovery. Most of the genes already reported to be associated with drug resistance are shared between all the surrogate strains and *M. tuberculosis*. We consider that *M. aurum* might be used in high-throughput screening for tuberculosis drug discovery. We also highly recommend the use of different model species during the drug discovery screening process.

**Electronic supplementary material:**

The online version of this article (doi:10.1186/s12864-017-3924-y) contains supplementary material, which is available to authorized users.

## Background

The world health organization (WHO) has predicted that nearly 1 billion people will have become infected with *Mycobacterium tuberculosis* between 2000 and 2020 and that tuberculosis (TB) will have cost the lives of 35 million people. New drugs are desperately needed to combat this disease [1, 2]. Traditional drug development approaches have previously provided effective drugs: rifampin as a first line drug in 1963 and fluoroquinolones used as a second line drug in 1983.

We are already in the “post antibiotic era” characterized by the worldwide distribution of multi-drug - resistant (MDR) and extremely drug resistant (XDR) *M. tuberculosis* strains [2]. International efforts have boosted anti-TB drug discovery over the last decade. Several chemical libraries of synthetic or natural compounds have been screened to identify new drugs for shorter and more effective TB treatment regimens [3–7]. This has so far led to the discovery and approval (in 2012) of only one new anti-TB drug with a new scaffold, bedaquiline, identified by screening using *M. smegmatis* as surrogate [8–11]. The use of bedaquiline however is limited to treatment of severe MDR TB cases due to severe side effects observed in animal models and an increased number of deaths observed during the first clinical trials published on the group of patients receiving bedaquiline in addition to the recommended W.H.O. treatment at that time [12].

Fast growing mycobacterial species show natural resistances to several drugs due to inactivating enzymes an impermeable cell wall. The use of fast growing non-pathogenic mycobacteria with different susceptibility/resistance profiles than *M. tuberculosis* may result in the discovery of new antibacterial molecules and natural resistance mechanisms in *M. tuberculosis*. Such a strategy has led to the use of clavulanic acid to circumvent resistance to some beta-lactams. This concept could be developed for other natural resistance mechanisms encountered in bacterial species. In this study, one of our major goals was to thoroughly profile the drug resistance profile and obtain the first complete genome sequence of the species *Mycobacterium aurum* ATCC23366. This species has already been proposed as a surrogate for *M. tuberculosis* drug resistance studies [13]. *M. aurum* is a fast-growing bacterium that does not form aggregates, thus facilitating many measurements, in particular precise enumeration. No complete genome sequence of this bacteria has been available until now [13, 14]. Here we present the first complete annotated genome sequence of *M. aurum*. In addition, we compared the entire coding capacity of each surrogate species used for *M. tuberculosis* drug screening to identify their common and specific metabolic pathways.

## Results

### Drug resistance profiling

We determined the MIC for *M. tuberculosis* H37Rv, *M. smegmatis* mc2 155, *M. marinum* ATCC BAA-535*,* and *M. aurum* ATCC23366 for the following antibiotics: kanamycin, amikacin, rifampin, isonizid, ethambutol, streptomycin, vancomycin clarithromycin, linezolid, and ofloxacin. The result is summarized in Table 1. We considered MICs only differing by two-fold to be equal as the rezasurine method is a colorimetric assay based on serial dilutions. The MIC was similar between *M. tuberculosis* and *M. aurum* for six out of the 12 antibiotics tested (Table 1).

**Table 1:**
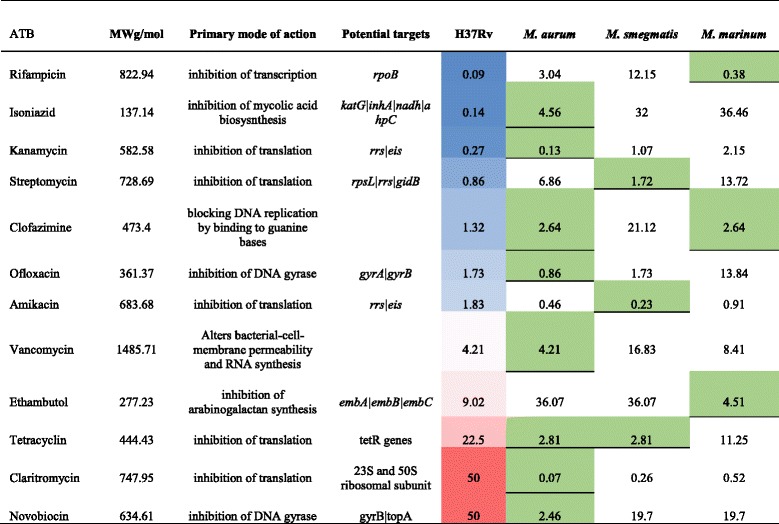
Drug resistance profile in uM

For *M. tuberculosis* low and high MICs are indicated in color gradient from blue to red. The green color reflects the lowest MIC when comparing *M. aurum*, *M. smegmatis* and *M. marinum*

### The genome of *M. aurum*

**Fig. 1:**
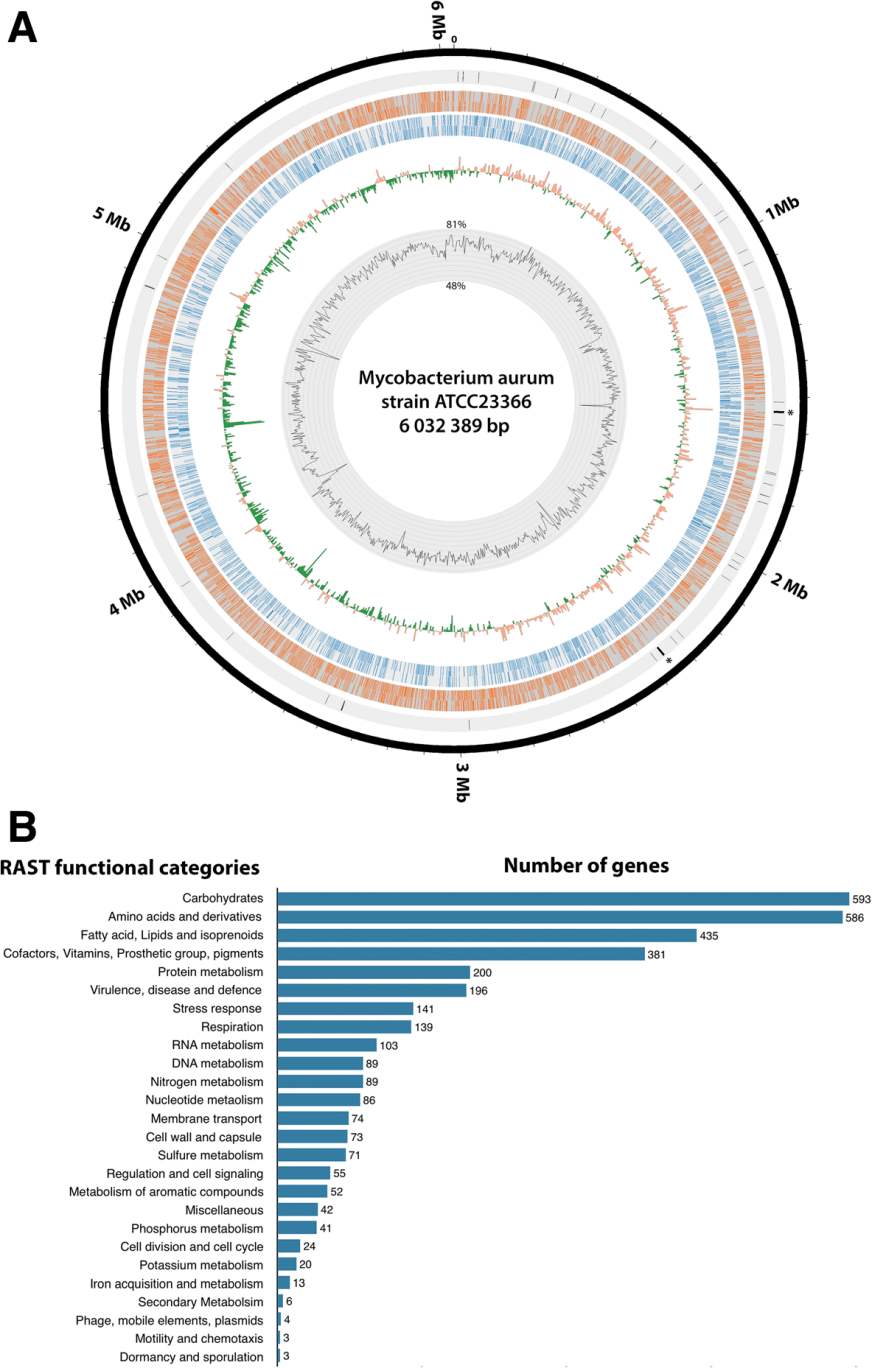
Characterization of the complete genome sequence of *M. aurum,* strain ATCC23366. **a** Circular map of *M. aurum* genome sequence using circos [35]. The *first* and *second rings* represent the GC content and GC skew within a window size of 5 kb, respectively. The *third* and *fourth rings* with *blue* and *red bars* represent the distribution of genes in the forward and lagging strands, respectively. The *fifth ring* shows the distribution of rRNA and tRNA. (*) 5S, 16S and 23S. **b** Histogram of the number of annotated genes in each category using RAST server

The sequencing of the *M. aurum* genome and its assembly led to a complete genome sequence of 6,032,389 bp (Fig. 1a) with a GC content of 68.5% [min = 48%, max = 81.3%] (Fig. 1a). RAST annotation in combination with tRNAscan-SE and RNammer led to the identification of 5750 coding sequences, 46 tRNA, and two rRNA operons (Additional file 1). The genome annotation showed that the coding capacity of the *M. aurum* genome is 92%. Among the 5750 coding sequences, 53% are transcribed from the positive strand and 47% from the lagging strand (Fig. 1a).

### Comparative genomics

**Fig. 2:**
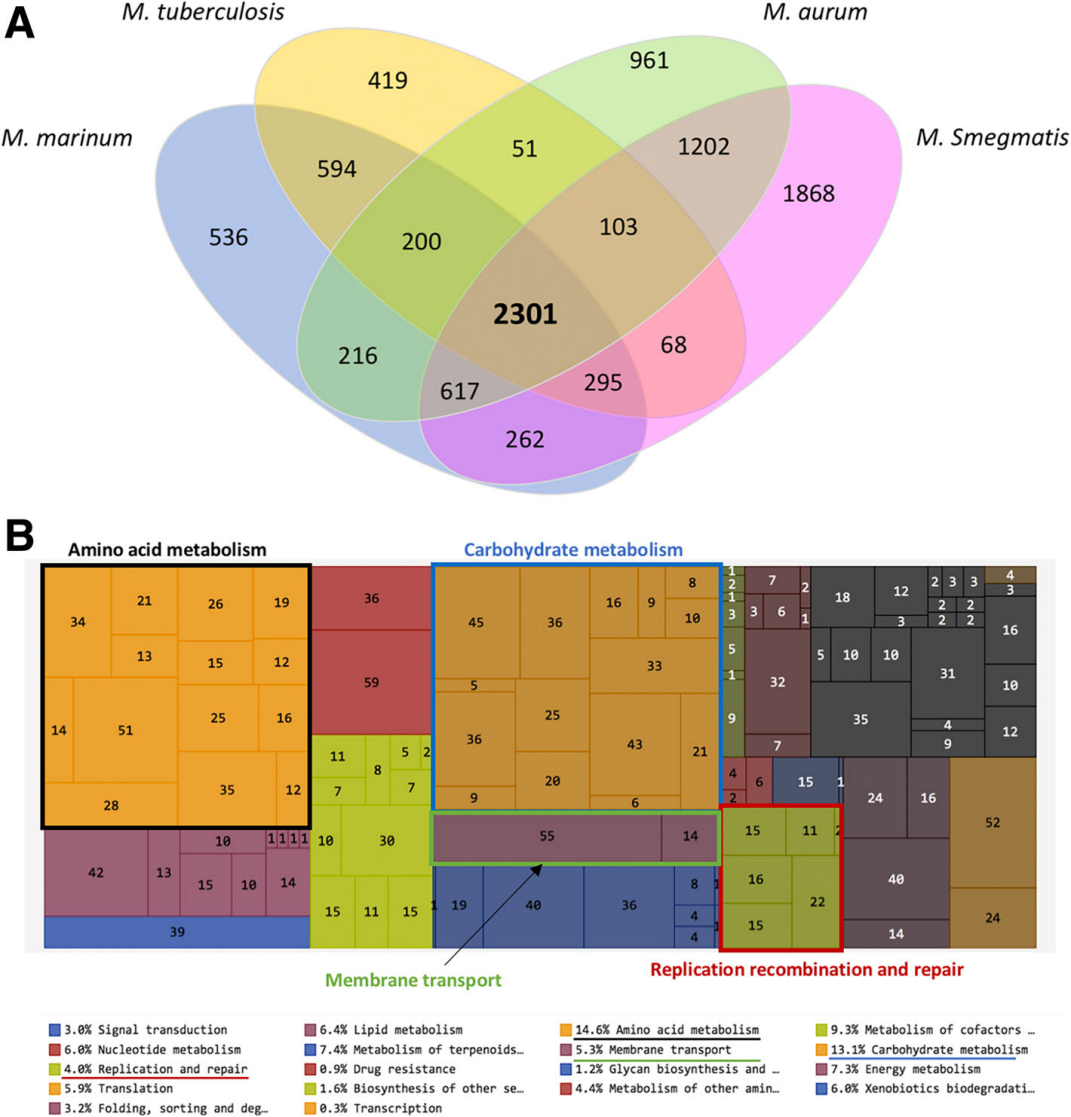
Comparative genomic analyses of *M. aurum*, *M. smegmatis*, *M. marinum* and *M. tuberculosis*. **a** Venn diagram based on the Reciprocal Best Hit result. The numbers correspond to the number of orthologous genes shared between the compared genomes. **b** Treemap of the KEGG metabolic pathways of the shared orthologous genes. Each *color* corresponds to one metabolic pathway. The different *boxes* correspond to the different sub-categories inside each KEGG category. The numbers correspond to the number of genes in each sub-category

**Fig. 3:**
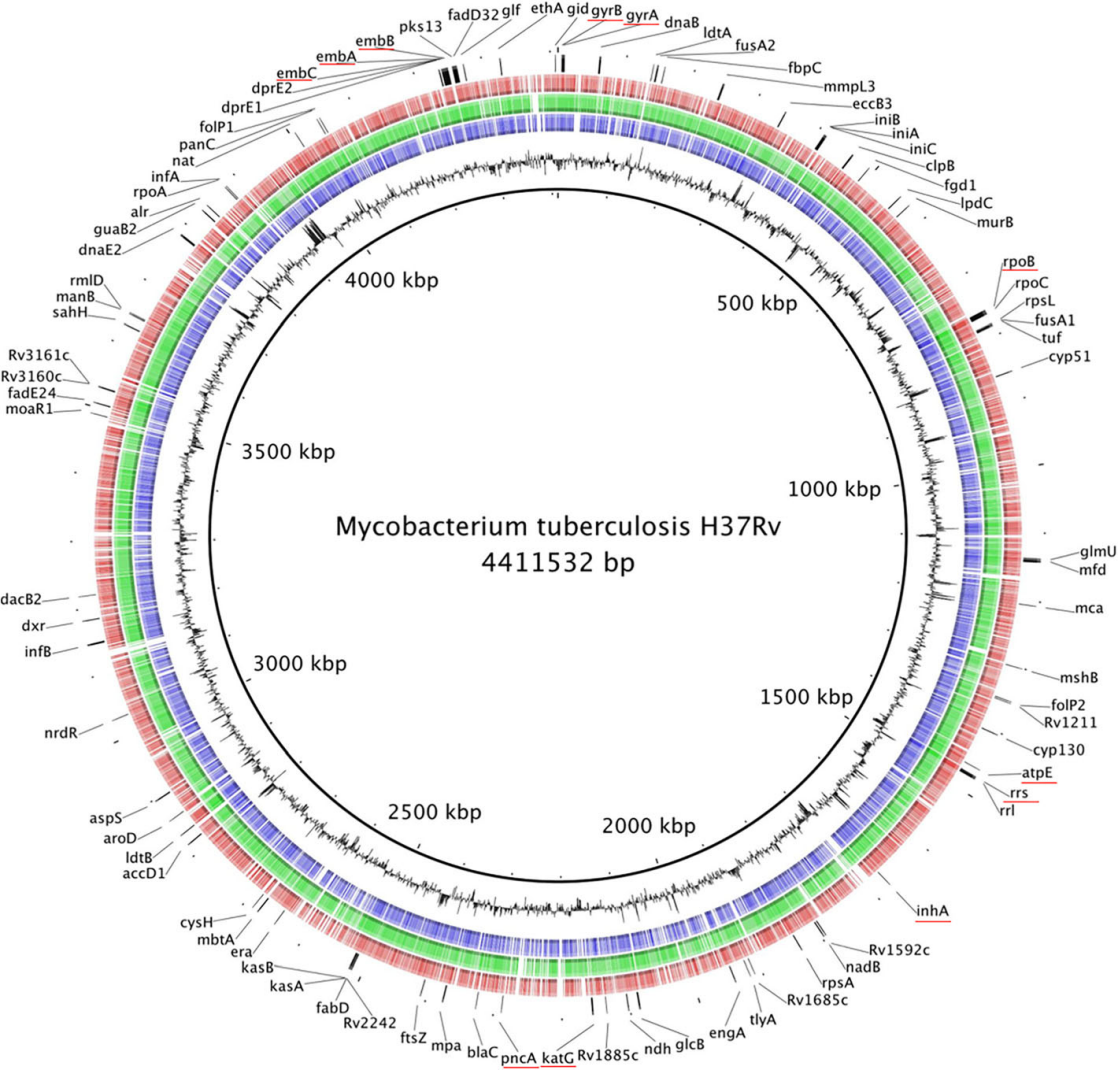
Whole genome alignment of the model species relative to *M. tuberculosis*. Each ring corresponds to one genome. *Blue ring*, *M. marinum*; *Green ring*, *M. smegmatis*; *red ring*, *M. aurum*. The indicated genes correspond to those that are already reported to be associated with mycobacterium drug resistance

When aligned, the average similarity between the analyzed genomes is equal to 80% (Fig. 2a). Using the Reciprocal Best Hit (RBH) strategy, we found that all genomes shared 2301 orthologous genes representing the core genome. This set of genes were classified according to the KEGG functional classification (Fig. 2b, Additional file 2). Most of these genes belong to two main categories: amino acid metabolism (14.6%) and carbohydrate metabolism (13.1%). We found that *M. aurum*, *M smegmatis* and *M. marinum* share 354, 466, and 1089 additional orthologous genes, respectively. The genome of *M. aurum* shares 4223 orthologous genes with that of *M. smegmatis* and 3334 genes with that of *M. marinum*. We analyzed the distribution of drug resistance-related genes among the genomes of this study. Most of the genes already known to be associated with drug resistance in *M. tuberculosis* are among the list of 2301 shared orthologues (Fig. 3). *M. tuberculosis* has 419 specific genes relative to the various species included in this study.

### Exclusive and shared genes with *M. tuberculosis* H37Rv

**Fig. 4:**
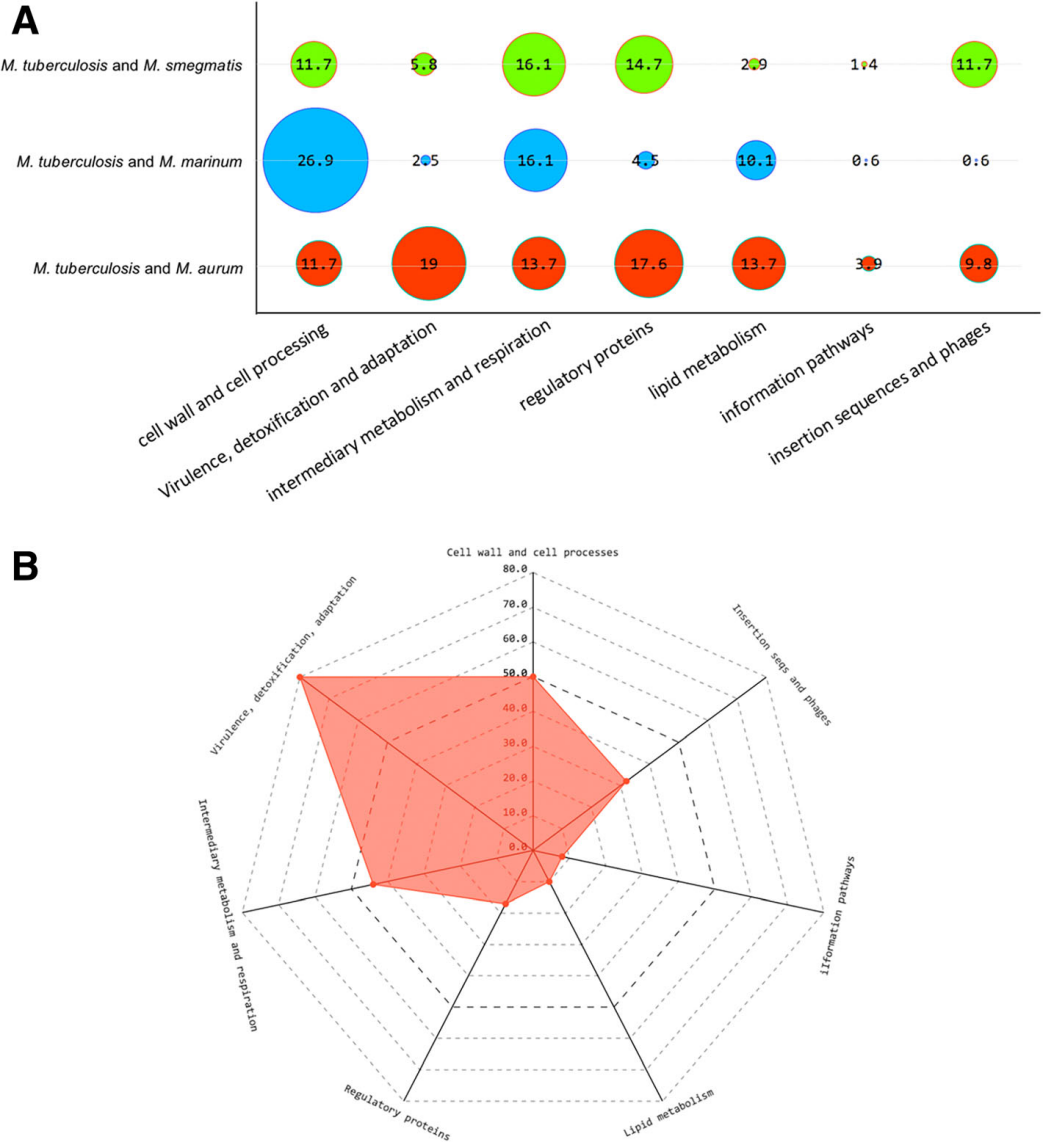
Shared metabolic pathways based on the specific identified genes between the surrogate model species and *M. tuberculosis* H37Rv. **a** *dot plot* showing the data corresponding to Tuberculist functional categories of the genes that are specific to each model species relative to *M. tuberculosis*, strain H37Rv. The number inside each *circle* represent the proportion of each category. **b** radar chart showing the number of each genes in each Tuberculist functional category relative to the 419 specific genes to *Mycobacterium tuberculosis*, strain H37Rv. For graphical convenience, in both (**a**) and (**b**) graphs, the data relative to genes coding for unknowing functions are not shown. For more details refer to Additional files 1, 2, 3, 4, 5, and 6

Each of the surrogate species included in this study shared a set of specific genes with *M. tuberculosis*, strain H37Rv. *M. marinum* shares the highest number with 594 genes (Additional file 3), followed by *M. smegmatis* with 68 genes (Additional file 4) and finally *M. aurum* with 51 genes (Additional file 5). Functional classification grouping using Tuberculist shows that the list of genes that are specific between *M. tuberculosis* and *M. aurum* are mainly part of the virulence, detoxification, adaption (19%) and regulatory proteins (17%) categories. Regarding *M. marinum*, the shared genes with *M. tuberculosis* belong mainly to the cell wall and cell processing category (26.9%). Finally, for *M. smegmatis*, 16.1% of the genes belong to the intermediate metabolism and respiration (Fig. 4a). Among the 419 genes that are exclusive to *M. tuberculosis* (Fig. 4b, Additional file 6), 80 and 50 genes belong to the virulence, detoxification, adaptation and cell wall and cell processes categories, respectively.

## Discussion

Tuberculosis was declared a global emergency by the WHO in 1993. Nevertheless, this infectious disease is still one of the most devastating bacterial diseases worldwide, with high rates of morbidity and mortality, and of increasing concern related to the emergence of MDR and XDR strains [15, 16]. Rational development of new anti-TB agents will benefit from the use of new approaches to understand the genetics and physiology of *M. tuberculosis*. The availability of the genome sequence of *M. tuberculosis* [17] and powerful genetic tools has provided valuable information about some potential drug targets. *M. marinum* [18] and *M. smegmatis* [19] are already used as model species to study the genetics and physiology of *M. tuberculosis*, and for drug discovery. The use of a new model strain for drug discovery could help identify to new targets and pathways involved in mycobacterial drug resistance. *M. aurum* is a fast-growing mycobacterium with a doubling time of ~3 h and does not require biosafety level 3 for its manipulation. When cultivated in liquid media, *M. aurum* bacilli do not aggregate, enabling more precise cell counting and optical density measurements. Five of the 12 antibiotic molecules tested in this study (Table 1) showed a similar MIC between *M. aurum* and *M. tuberculosis*, H37Rv. *M. aurum* had the closest MIC profile to *M. tuberculosis* H37Rv relative to the other surrogate strains tested in this study and could thus be considered also as a potential surrogate for drug discovery. *M. aurum* has a larger genome than *M. tuberculosis*, with an exact size of 6,032,389 bp (Fig. 1a) similar to those of *M. marinum* (6.7 Mb) [18] and *M. smegmatis* (~7 Mb) [19]. Our study complement the work of Phelan et al., 2015 [14] by presenting the first reported complete genome sequence of *M. aurum*. More than 65% of the genes identified using RAST were annotated. By comparing the coding capacity of all strains analyzed in this study, a core set of 2301 genes was defined (Fig. 2a). This number is in agreement with other studies [19, 20] that compared different Mycobacterial species to *M. tuberculosis*. By taking into account this observation and the MIC profile of *M. aurum*, this species can be used as a valuable model to study *M. tuberculosis* growth inhibitors. Most of the core genes are involved in amino-acid and carbohydrate metabolism. Genes belonging to these categories play a major role in the survival of the bacteria and some may represent valuable targets for drug discovery. In addition, 5.3% of the core genes encode membrane transport proteins, including the MmpL and ESX secretion system protein families, including the type VII secretion system known as ESX-3. ESX-3 is already known to be essential for mycobacterial growth in vivo. Proteins that are part of the different ESX secretion systems are potential targets for drug development and discovery. Amongst the genes already reported to be directly associated or not with *M. tuberculosis* drug resistance, the fact that 86 selected genes (Fig. 3) are shared between all model species and H37Rv confirm their potential utility for the discovery of anti-TB drugs However, sharing the same list of genes does not necessarily lead to the same drug tolerance profile due to the influence of other factors in drug resistance. In addition, the cell walls of each model strain possess their own unique characteristics*.* The list of 419 genes that are exclusive to *M. tuberculosis* (Additional file 5), strain H37Rv represent a potential set of drug targets. In fact, part of the virulence, detoxification, adaptation category we found genes encoding toxin and antitoxin (TA) proteins. It has been proposed that activation of TA systems could facilitate bacterial survival until conditions become more favourable [21, 22]. The exploitation of TA systems as an antibacterial strategy via artificial activation of the toxin has been proposed and has considerable potential [23, 24]. Among these specific genes, we found also genes involved in molybdenum cofactor biosynthesis. Inhibiting enzymes involved in cell wall and molybdenum cofactor biosynthesis represent a promising strategy for drug discovery [25–27].

To conclude, the origin of the differences in the MIC observed between the different strains must be further investigated to better characterize the mode of action of some anti-TB drugs and define new pathways involved in mycobacterial drug resistance. The list of genes that are specific to *M. tuberculosis* when comparing to the three other surrogate species included in this study, must be investigated as they will now be directly targeted when screening for new compounds. Finally, while *M. aurum* seems to have a minimum inhibitory concentration profile closer to *M. tuberculosis* based on the antibiotics used in this study, we believe that basing the drug screening on only one surrogate species could lead to exclusion of interesting compound that could be chemically modified or combined to other molecules to increase their efficiency and specificity again *M. tuberculosis*. Finally, we propose that the resazurin assay, which is a colorimetric assay scalable to high-throughput screening, and considering the fact that *M. aurum* does not form clumps, it might be a better choice in this case.

## Conclusion

Here, we report the first complete genome sequence of *Mycobacterium aurum* ATCC23366, an environmental mycobacterium that can also grow in the gut of humans and animals as part of their microbiota. This species shows a comparable resistance profile to that of *M. tuberculosis* for several anti-TB drugs. The availability of the complete annotated genome sequence of *M. aurum* will make it more useful as a surrogate for mycobacterial drug discovery. The comparison between *M. aurum* and those surrogate species most used for the discovery of anti-TB drugs, suggests that it may represent a promising model strain as it shares more than 2300 genes, representing the core genome, with the model species included in this study. In addition, most of the genes already reported to be associated with drug resistance are shared between all the surrogate species studied and *M. tuberculosis*. The list of genes exclusive to *M. tuberculosis* could be further explored to better define good targets for drug discovery studies.

## Methods

### Cultivation of mycobacterial species and genomic DNA extraction

We used the Mueller Hinton media for culturing *M. smegmatis* mc2 155 and *M. aurum* ATCC23366 as each of the Mycobacterial strains used in this study have specific in vitro growth requirements when determining the minimum inhibitory concentration. *M. marinum* ATCC BAA-535 was cultured in Mueller Hinton Broth supplemented with 10% OADC. *M. tuberculosis* strain H37Rv was grown in Middlebrook 7H9 broth (Difco) supplemented with 10% ADC and 0.05% Tween 80.

### DNA extraction

The protocol of genomic DNA extraction of *M. aurum* is detailed in Kaser et al., 2010 [28].

### Drug resistance profiling using the microdilution Resazurin assay (MRA)

The assays were carried out as described by Banfi et al., 2003 (www.pacb.com) with modifications. The microdilution test was performed in 96-well plates. Two-fold dilutions of each drug were prepared in the test wells in complete 7H9 and Sauton broth for a final concentration from 10 down to 0.0048 μg/ml). Five microliters of each bacterial suspension were added to 100 uL drug-containing culture medium. Control wells were prepared using culture medium only and the bacterial suspension. For *M. tuberculosis*, the plates were sealed and incubated for seven days at 37 °C. *M. aurum* and *M. smegmatis* were cultivated at 37 °C overnight. For *M. marinum*, the bacterial culture was incubated at 33 °C during 4 days. After this incubation period, 30 ul of 0.01% resazurin solution was added to each well. The plates were incubated at their respective optimal temperature for an additional 24 h. The MIC was determined as the lowest concentration of antimicrobial agent at which no growth was observed. Blue wells are due to the absence of growth whereas pink wells reflect the presence of growing bacteria. All experiments were performed in triplicate for each mycobacterial species and for each drug tested in this study. The total number of viable bacteria was determined from the control wells after plating on 7H11 agar plates.

### *M. aurum* genome sequencing and assembly

A Pacific Biosciences RSII system (PacBio, University of Washington, Seattle, WA) was used [29] to obtain the complete genome sequence of *M. aurum*. A 3 to 20 kb library of this strain was prepared and sequenced using C2 chemistry kits on one single-molecule real-time (SMRT) cell with a 90-min collection protocol, achieving an average genome coverage of >100× for the strain. The 3 to 20- kb continuous-long-read (CLR) data were assembled de novo using the PacBio Hierarchical Genome Assembly Process 2 (HGAP2)/Quiver software package. The 5′ and 3′ 10 kb regions were investigated and aligned using Geneious (version 7.1) to identified primer sequence and overlapping regions.

### *M. aurum* genome annotation

The complete genome sequence of *M. aurum* was submitted to the online Rapid Annotation Subsystems Technology (RAST) server [30, 31]. The tRNA and rRNA genes were verified using the tRNAscan-SE search server [32] and RNammer [33]. This genome annotation was used to identify the gene dnaA, which was used as the first gene of the *M. aurum* genome. The last annotated gene was rpmH gene. This genome manipulation was done using Geneious (version 7.1). The annotation of *M. aurum* using RAST and Prokka (data not shown), did not lead to the identification of any plasmid coding sequences. In addition, we analyzed the genome sequence using PlasmidFinder (version 1.3) that also showed no plasmid sequences in the *M. aurum* genome.

### Comparative genomics

All open reading frames (ORFs) annotated in the genome sequences of *M. tuberculosis* H37Rv, *M. smegmatis*, and *M. marinum* were retrieved from the National Center for Biotechnology Information (NCBI) website. These ORFs were combined with those identified in the genome sequence of *M. aurum* and compared using a Reciprocal Best Hit (RBHs) strategy with an E-value threshold of up to 100 to maximize our chances of finding homologs. We used UBLAST, as implemented in the sequence analysis multitool USEARCH (version 8.1.1812). Several python scripts were written to compare and manage the generated data. Data visualization was made using BRIG [31].

### Functional classification using Tuberculist and KEGG pathways

All functional categories were obtained using the Kyoto Encyclopedia of Genes and Genomes (KEGG) [34] and Tuberculist (http://tuberculist.epfl.ch/). A python script was written to retrieve the Tuberculist and KEGG functional categories relative to *M. tuberculosis* H37Rv. Several python scripts were written to visualize the different data as a treemap.

## Additional files

Additional file 1: *Mycobacterium aurum* genome annotation. (XLSX 752 kb)

Additional file 2: List of orthologous genes with their corresponding functions. (XLSX 243 kb)

Additional file 3: List of specific genes shared between *M. tuberculosis* H37Rv and *M. smegmatis*. (XLSX 20 kb)

Additional file 4: List of specific genes shared between *M. tuberculosis* H37Rv and *M. marinum*. (XLSX 49 kb)

Additional file 5: List of specific genes shared between *M. tuberculosis* H37Rv and *M. aurum*. (XLSX 24 kb)

Additional file 6: List of specific genes to *M. tuberculosis* H37Rv after comparing to all surrogate strains. (XLSX 35 kb)

## Abbreviations

ADC: Albumin Dextrose Catalase
CLR: Continuous Long Reads
*M. aurum*: *Mycobacterium aurum*
*M. marinum*: *Mycobacterium marinum*
*M. smegmatis*: *Mycobacterium smegmatis*
*M. tuberculosis*: *Mycobacterium tuberculosis*
MDR: Multidrug Resistant
MIC: Minimum inhibitory concentration
OADC: Oleic Albumin Dextrose Catalase
TA system: Toxin-Antitoxin system
TB: Tuberculosis
WHO: World health organization
XDR: Extensively Drug-Resistant
